# Reproducibility of Ablated Volume Measurement Is Higher with Contrast-Enhanced Ultrasound than with B-Mode Ultrasound after Benign Thyroid Nodule Radiofrequency Ablation—A Preliminary Study

**DOI:** 10.3390/jcm9051504

**Published:** 2020-05-16

**Authors:** Simone Schiaffino, Francesca Serpi, Duccio Rossi, Valerio Ferrara, Ciriaco Buonomenna, Marco Alì, Lorenzo Monfardini, Luca Maria Sconfienza, Giovanni Mauri

**Affiliations:** 1 Radiology Unit, IRCCS Policlinico San Donato, San Donato Milanese, 20097 Milan, Italy; marco.ali90@gmail.com (M.A.); schiaffino.simone@gmail.com (S.S.); 2 Post-graduate School in Radiodiagnostics, Università degli Studi di Milano, 20122 Milan, Italy; fr.serpi@gmail.com (F.S.); valerio.ferrara@unimi.it (V.F.); ciriaco.buonomenna@unimi.it (C.B.); 3 Unit of Diagnostic Imaging and Stereotactic Radiosurgery, C.D.I. Centro Diagnostico Italiano S.p.A., 20147 Milan, Italy; 4 Radiology Unit, Fondazione Poliambulanza, 25124 Brescia, Italy; lorenzo.monfardini@poliambulanza.it; 5 IRCCS Istituto Ortopedico Galeazzi, 20161 Milan, Italy; io@lucasconfienza.it; 6 Department of Scienze Biomediche per la Salute, Università degli Studi di Milano, 20122 Milan, Italy; 7 Division of Interventional Radiology, European Institute of Oncology, IRCCS, 20141 Milan, Italy; vanni.mauri@gmail.com; 8 Department of Oncology and Hemato-Oncology, Faculty of Medicine and Surgery University of Milan, 20122 Milan, Italy

**Keywords:** thyroid nodule, radiofrequency ablation, ultrasonography, contrast-enhanced ultrasound, observer variation

## Abstract

The reproducibility of contrast-enhanced ultrasound (CEUS) and standard B-mode ultrasound in the assessment of radiofrequency-ablated volume of benign thyroid nodules was compared. A preliminary study was conducted on consecutive patients who underwent radiofrequency ablation (RFA) of benign thyroid nodules between 2014 and 2016, with available CEUS and B-mode post-ablation checks. CEUS and B-mode images were retrospectively evaluated by two radiologists to assess inter- and intra-observer agreement in the assessment of ablated volume (Bland–Altman test). For CEUS, the mean inter-observer difference (95% limits of agreement) was 0.219 mL (-0.372–0.809 mL); for B-mode, the mean difference was 0.880 mL (-1.655–3.414 mL). Reproducibility was significantly higher for CEUS (85%) than for B-mode (27%). Mean intra-observer differences (95% limits of agreement) were 0.013 mL (0.803–4.097 mL) for Reader 1 and 0.031 mL (0.763–3.931 mL) for Reader 2 using CEUS, while they were 0.567 mL (-2.180–4.317 mL, Reader 1) and 0.759 mL (-2.584–4.290 mL, Reader 2) for B-mode. Intra-observer reproducibility was significantly higher for CEUS (96% and 95%, for the two readers) than for B-mode (21% and 23%). In conclusion, CEUS had higher reproducibility and inter- and intra-observer agreement compared to conventional B-mode in the assessment of radiofrequency-ablated volume of benign thyroid nodules.

## 1. Introduction

Thyroid nodules are a common occurrence in the general population, with a prevalence between 20% and 70% [1,2,3,4,5]. The vast majority are benign and incidentally detected at ultrasound (US) examination. Although they are normally asymptomatic and can be managed conservatively (i.e., clinical and US follow-up), some of them may require treatment because of cosmetic reasons, subjective compressive symptoms (for compression on structures around the thyroid gland), or for an abnormally increased function of the nodule [1,6].

Surgery has been traditionally the main treatment option for thyroid nodules, carrying however several drawbacks, such as scars, need for general anesthesia, and risk of induced hypothyroidism [7]. Therefore, minimally invasive image-guided thermal ablation techniques have been increasingly applied in the last years in order to reduce the invasiveness of treatment in patients with benign thyroid nodules. Among image-guided thermal ablation techniques, laser and radiofrequency ablation (RFA) are the most commonly used [8,9]. Some guidelines and consensus documents suggested the application of image-guided thermal ablation as an alternative to surgery in patients with symptomatic thyroid nodules, even as first-line treatment [7,10,11].

Although thermal ablation is a safe and effective procedure, nodule regrowth can occur during follow-up, with a rate of 5.5% and 9% for RFA and laser, respectively reported in literature [12,13,14].

To find early signs of a potential future nodule regrowth would be of relevant clinical value. Usually, technique efficacy is defined as a volume reduction >50% of the initial volume and is evaluated at one year after treatment [15,16]. Recently, some authors introduced initial ablation ratio (IAR) as a quantitative early indicator correlated with volume reduction ratio during follow-up [15]. In their paper, Sim et al. evaluated the IAR identifying the ablated area on standard B-mode ultrasound images [16]. However, some time margins of the ablated area can be difficult to precisely delineate on B-mode ultrasound. In this scenario, contrast-enhanced ultrasound (CEUS) is largely used after ablation to better identify the ablated area and could improve IAR definition in thyroid ablations [17,18,19,20].

The aim of this study was to compare the reproducibility of CEUS and B-mode and to evaluate inter- and intra-observer agreement in the assessment of the radiofrequency-ablated volume of benign thyroid nodules, comparing CEUS and B-mode.

## 2. Experimental Section

### 2.1. Patients

The local ethics committee approved this study (San Raffaele Hospital Ethics Committee, registry number 63/INT/2020). This study was partially supported by Ricerca Corrente funding from Italian Ministry of Health to IRCCS Policlinico San Donato. Due to the retrospective nature of this study, specific informed consent was waived.

We retrospectively assessed consecutive patients with predominantly solid nonfunctioning thyroid nodules treated with RFA in a single center between January 2014 and December 2016. All patients declined or were not eligible for surgery. Benign proven thyroid nodules causing compressive symptoms and cosmetic concerns were considered for treatment.

Eligibility criteria for inclusion in the study were patients who underwent RFA of benign thyroid nodules and whose follow-up included CEUS and B-mode evaluation one year after treatment.

### 2.2. Preablation Assessment and Procedure

All patients had a cytologically proven benign, TI-RADS 2 category (benign findings, according to the Thyroid Imaging Reporting and Data System) [21] thyroid nodule determining pressure symptoms and/or cosmetic problems.

All procedures were performed under local anesthesia and conscious sedation by one interventional radiologist with 10 years of experience in image-guided percutaneous ablations.

Under the guidance of a high-resolution linear transducer (L3-12a linear transducer, RS80A with Prestige US System—Samsung Medical Imaging, Seoul, South Korea), we used an internally cooled, 18G electrode with a 0.5–1.5 cm active tip (AMICA, HS Hospital Service, Aprilia, Italy) and a free-hand “moving-shot” technique [22].

### 2.3. Follow-Up Examinations

All follow-up US scans were taken one, six, and twelve months after ablation in the same center by the same interventional radiologist who performed RFA, using a high-frequency linear probe (L3-12a linear transducer, RS80A with Prestige US System—Samsung Medical Imaging, Seoul, South Korea). Technical parameters, including gain adjustment, field of view, and transmission focusing, were optimized for thyroid imaging.

Two perpendicular images of the largest ablated area were acquired for each treated nodule with CEUS and B-mode.

CEUS evaluation was performed the same day of the B-mode scan, after intravenous administration of 4.8 mL of Sonovue (Bracco Imaging, Milan, Italy) with the same US scanner and linear probe, using a dedicated preset.

### 2.4. Radiofrequency-Ablated Volume Assessment

Two physicians (V.F. and C.B., with 5 and 6 years of experience in thyroid imaging respectively) retrospectively reviewed US images of the last check (one year after treatment) on the Picture Archiving and Communication System. They measured the ablated volume on gray-scale B-mode images in a first session and on CEUS images in a second session, after two weeks and randomization of the cases. To assess intra-observer agreement, a second reading session for both B-mode and CEUS scans was performed one month after the first one.

All measurements were blindly taken in random order and freehandedly. Three orthogonal nodule diameters, including the largest diameter, were measured on both B-mode and CEUS images. Nodule volume was calculated using the equation V = πabc/6, where V is the volume, a is the longest diameter, and b and c are the other two perpendicular diameters.

### 2.5. Statistical Analysis

Inter- and intra-observer agreement was assessed by using the Bland–Altman test. Mean difference (bias) and the 95% limits of agreement (mean difference ± 1.96 standard deviation) were determined. Statistical analysis was performed using commercial software packages (IBM SPSS Statistics for Windows, version 26.0, IBM Corp., Armonk, NY, USA).

## 3. Results

A total of thirty-four patients were treated between January 2014 and December 2016, twenty-three of whom met the inclusion criteria and were included. Patient and nodule characteristics are detailed in Table 1.

### 3.1. Inter-observer Agreement

Regarding inter-observer agreement, CEUS showed a bias (95% limits of agreement) of 0.219 mL (-0.372 to 0.809 mL) and a coefficient of reproducibility (COR) of 0.593 mL (*p* = 0861). B-mode had a bias of 0.880 mL (-1.655 to 3.414 mL) and a COR of 2.558 mL (*p* = 0.115). CEUS showed a higher inter-observer reproducibility (85%) compared to B-mode (27%).

Reader 1 had higher mean volume estimation compared to Reader 2 using both CEUS and B-mode.

**Figure 1:**
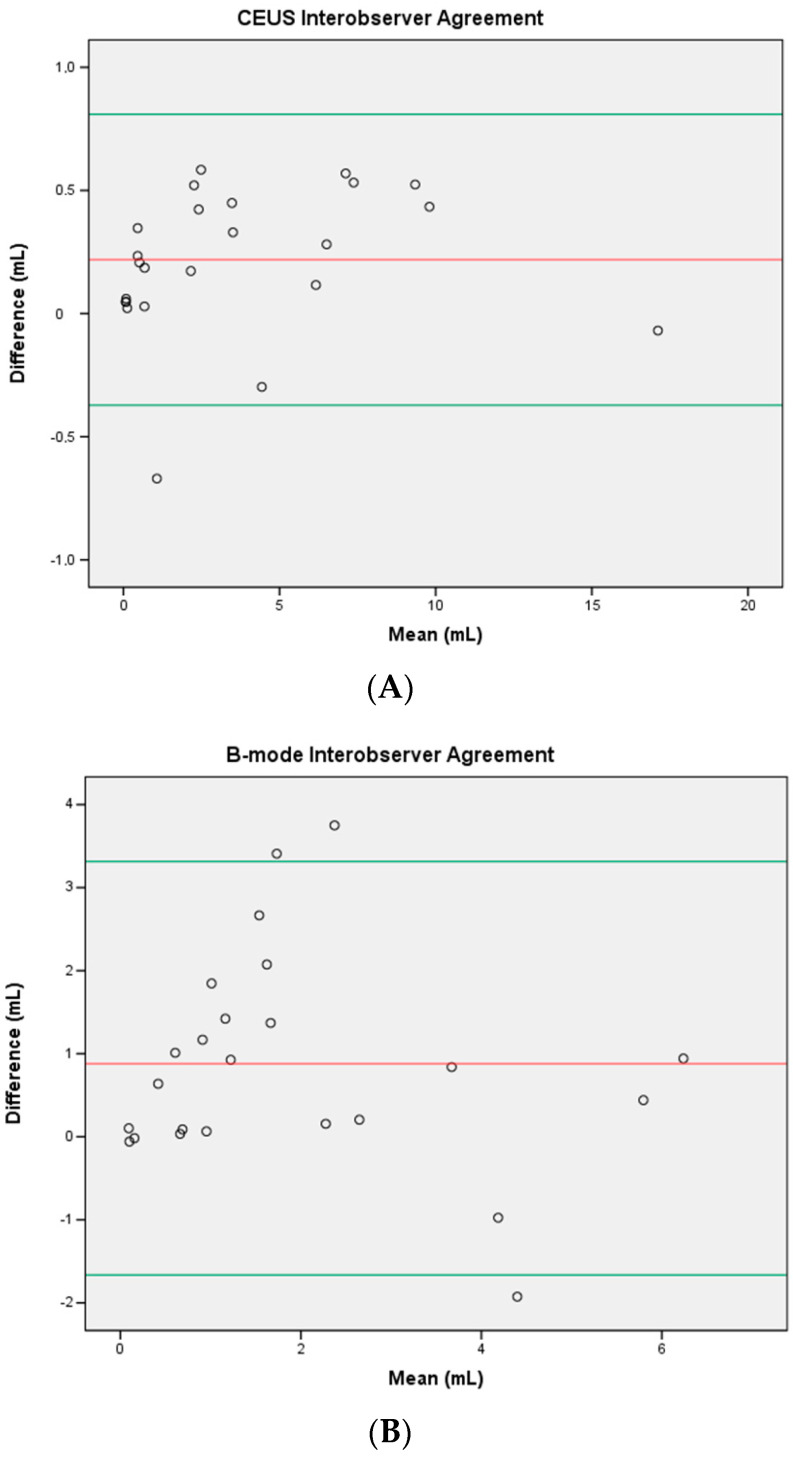
Bland–Altman plots showing inter-observer reproducibility of measurements for CEUS (**A**) and B-mode (**B**) assessments. The x-axes show the mean of the volume measurements, and the y-axes show the differences between the measurements. Red lines = mean difference between readers, and green lines = 95% (1.96 SD) limits of agreement.

Plots are shown in Figure 1.

### 3.2. Intra-observer Agreement

Intra-observer agreement was better for CEUS compared to B-mode for both the readers: a bias (95% limits of agreement) of 0.013 mL (0.803 to 4.097 mL) was found for Reader 1 assessing his intra-observer reproducibility assessing ablated volume using CEUS, and 0.031 mL (0.763 to 3.931 mL) for Reader 2. For CEUS, COR was 0.159 mL for Reader 1 (*p* = 0.992) and 0.190 mL for Reader 2 (*p* = 0.980), and reproducibility was 96% and 95%, respectively.

The ablated volume assessed with B-mode showed a bias of 0.567 mL (-2.180 to 4.317 mL) and 0.759 mL (-2.584 to 4.290 mL) for Readers 1 and 2, respectively. For B-mode, COR was 2.203 mL for Reader 1 (*p* = 0.286) and 2.397 mL for Reader 2 (*p* = 0.169), and reproducibility was 21% and 23%, respectively.

**Figure 2:**
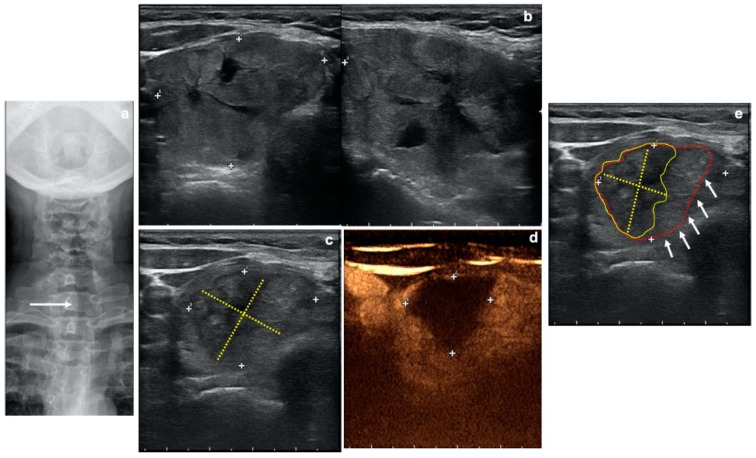
Case of a 56-year-old woman treated with radiofrequency ablation. *(***a**) Thoracic inlet X-ray showing a large retrosternal goiter with left deviation (white arrow) and indentation of the trachea. *(***b**) Two-planes gray-scale ultrasound (US) showing pretreatment evaluation of an almost completely solid right thyroid lobe nodule (Volume: 28.8 mL). *(***c**) Gray-scale US at 6 months after treatment showing volumetric reduction of the ablated nodule (yellow dotted lines = evaluation of the ablated area with B-mode US). *(***d**) Contrast-enhanced ultrasound (CEUS) at 6 months after treatment demonstrating the area of ablation as an area with lack of enhancement (white crosses = evaluation of the ablated area with CEUS). *(***e**) After CEUS-treated nodule evaluation, treatment results can be better reconsidered on the grey-scale US (c) showing real ablated nodule margins (yellow dotted lines and encircled area) as compared to previously measured margins (white arrows; red encircled area).

Plots are available as Supplementary Figures S1–4. Figure 2 shows a right nodule ablation results evaluation where B-mode US and CEUS are compared.

## 4. Discussion

Image-guided thermal ablation is becoming increasingly common as an alternative to surgery for the treatment of a variety of benign and malignant conditions [23,24,25,26,27,28,29,30,31,32]. When dealing with diseases in the neck, image-guided thermal ablations have been reported to be effective in the treatment of benign thyroid nodules, autonomously functioning nodules, metastatic lymph nodes, and recurrent thyroid cancers [9,12,33,34,35,36,37]. In the treatment of benign thyroid nodules, different techniques have been used, including laser and RFA, which are the most widely used techniques, and have been proven to be effective and with good results over time in terms of nodule volume reduction [31,33,34,38,39,40,41]. In order to evaluate the success of the procedure, it is crucial to follow treated nodules over time. In fact, some of them might regrow and need further ablation sessions.

US is the most widely used imaging modality for the assessment of thyroid nodules. With B-mode, a central hypoechoic area is considered as a direct sign of ablation zone (immediately after the procedure) and expression of tissue remodeling (during follow-up). From a recent study conducted with B-mode, the central ablated area can be used to determine a quantitative index to predict therapeutic success [16]. According to this study, IAR is the ratio of the ablated volume to total volume of the nodule. If IAR after RFA is <70%, the nodule is likely to regrow [16].

However, some limitations of US, such as low reproducibility and operator-depending performance and measuring, might reduce its accuracy in the evaluation of thyroid nodules’ ablated volume. Particularly, the ablated area can be difficult to clearly demarcate with B-mode, as it can appear as an isoechoic area in comparison with nonablated surrounding thyroid tissue. In this setting, CEUS could be helpful in achieving a better demarcation of the ablated area and to improve the reproducibility of the measurements. In fact, CEUS provides information regarding the vascularization of a human tissue and is widely used in the US assessment of ablations results in other organs [18,20,35,36,42]. Furthermore, it is applied in some centers to precisely delineate the ablated area in thyroid nodules treated with image-guided thermal ablation [34,37,43]. Notably, US contrast agents can be directly administered to complete a standard US examination and are extremely safe and well tolerated. In fact, US contrast agents are not excreted through the kidneys and can be safely administered to patients with renal insufficiency with no risk of contrast-related nephropathy or nephrogenic systemic fibrosis. There is no need for blood tests prior to injection, and there is no evidence of any effect on thyroid function, as they do not contain iodine. These contrast agents have a very low rate of anaphylactoid reactions (1:7000 patients, 0.014%), significantly lower than the rate with iodinated computer tomography (CT) contrast agents (35–95:100,000 patients, 0.035–0.095%), comparable to the rate of severe anaphylactoid reactions associated with gadolinium-based contrast agents at 0.001–0.01% [43].

In this study, we observed a significantly higher inter- and intra-observer reproducibility in measuring the ablated area with CEUS compared to those with B-mode. CEUS, in fact, better enhances vascularized tissue, which helps to clarify boundaries between viable and nonviable tissue. This could be helpful to obtain a more precise and reproducible measure of the ablated area. In our study, it helped in fact to reduce intra- and inter-observer variation. Some studies evaluated the inter-observer and intra-observer variations assessment of thyroid nodules with US [44,45,46]. All these studies were focused in evaluating the diagnostic performance of US in the diagnosis of malignancy among the investigated nodules, while, to the best of our knowledge, no study reported on the analysis of inter-observer and intra-observer agreement in the evaluation of the ablated area. Thus, our paper is the first to depict the potential role of adding CEUS to standard B-mode ultrasound to increase the reproducibility of the measurement of the ablated area after radiofrequency ablation of benign thyroid nodules.

This study has some limitations. First, it is a retrospective study, performed in a single center with a low number of cases. Larger prospective studies are necessary to confirm our preliminary data. Second, the real value of a precise evaluation of the ablated area is still under investigation, and its potential role in predicting a regrowth is still unproven. Therefore, the potential clinical value is still hypothetical. Third, the two readers had a quite similar experience in thyroid ultrasound, and the variability among operators with larger difference in experience should be further investigated.

## 5. Conclusions

US follow-up is crucial after RFA of benign thyroid nodules. CEUS, compared to conventional B-mode, better depicts the ablated area and for this reason reduces variation in its measurement, with a significantly higher inter- and intra-observer reproducibility. Thus, application of CEUS during the follow-up of patients treated with RFA for a benign thyroid nodule can be beneficial to achieve a better definition of the ablated area. Further studies on larger samples are needed to better understand the potential clinical value of these findings.

## Figures and Tables

**Table 1:** Patient and nodule characteristics.

***N***	23	
Females/Males	20/3	
Mean age (± standard deviation)	60 (±14) years	
Nodule location	Right	9
	Isthmic	3
	Left	11
Nodule mean volume before treatment (mL)	23.90 ± 17.2 (range 7.3–62.5)	
Mean ablated volume (± standard deviation) assessed with CEUS (mL)	Reader 1	3.947 ± 4.243
	Reader 2	3.729 ± 4.196
Mean ablated volume (± standard deviation) assessed with B-mode (mL)	Reader 1	2.441 ± 1.735
	Reader 2	1.562 ± 1.965

CEUS—contrast-enhanced ultrasound.

## Supplementary Materials

The following are available online at https://www.mdpi.com/2077-0383/9/5/1504/s1, Figure S1: Reader 1 CEUS, Figure S2: Reader 1 B-mode, Figure S3: Reader 2 CEUS, Figure S4: Reader 2 B-mode.
