# Supplementary material for: Reproducibility of Ablated Volume Measurement Is Higher with Contrast-Enhanced Ultrasound than with B-Mode Ultrasound after Benign Thyroid Nodule Radiofrequency Ablation—A Preliminary Study
Source: J Clin Med. 2020 May 16;9(5):1504. doi: 10.3390/jcm9051504 (PMC7291258; doi:10.3390/jcm9051504)
Supplement: Supplementary file 1 [file jcm-09-01504-s001.zip › jcm-764004-supplementary.docx]

Supplementary Materials


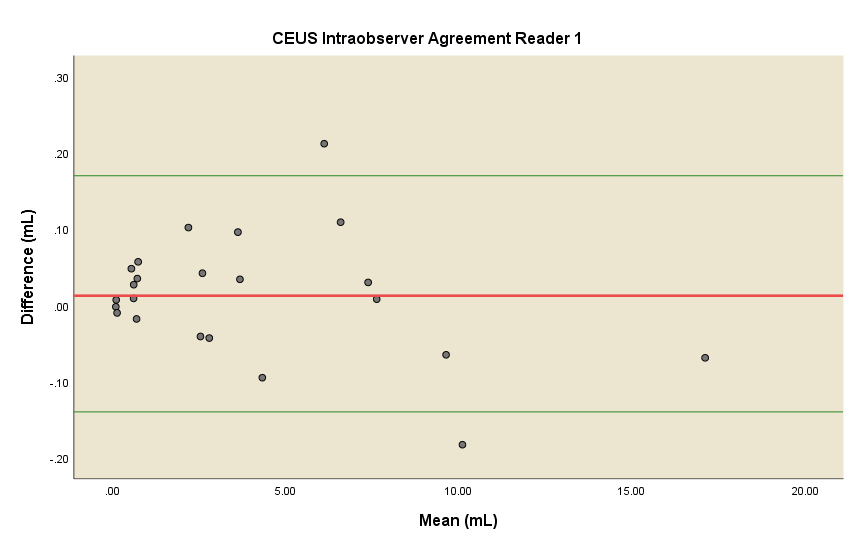


**Supplementary Figure 1.** Reader 1 CEUS.


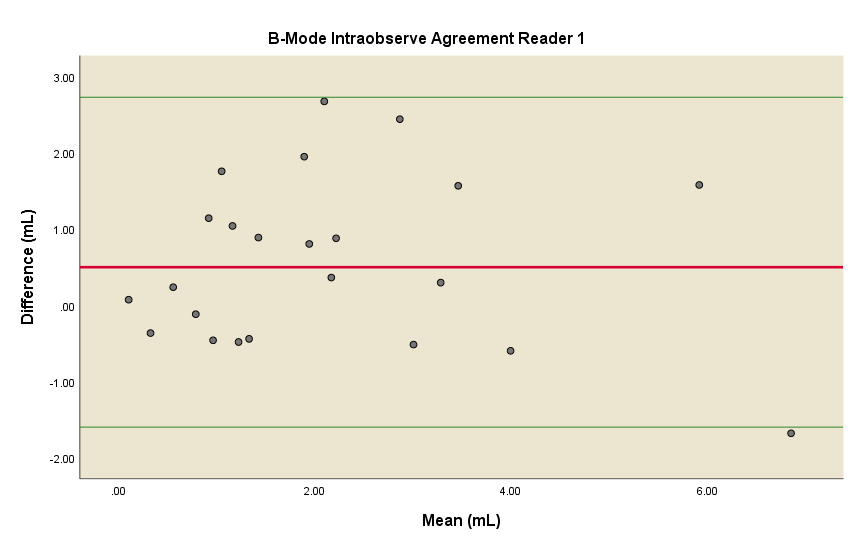


**Supplementary Figure 2.** Reader 1 B-mode.


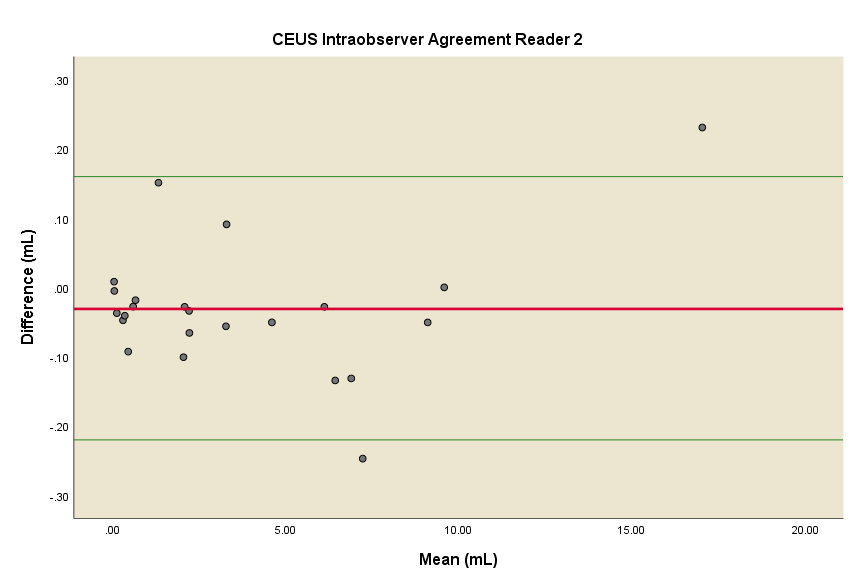


**Supplementary Figure 3.** Reader 2 CEUS.


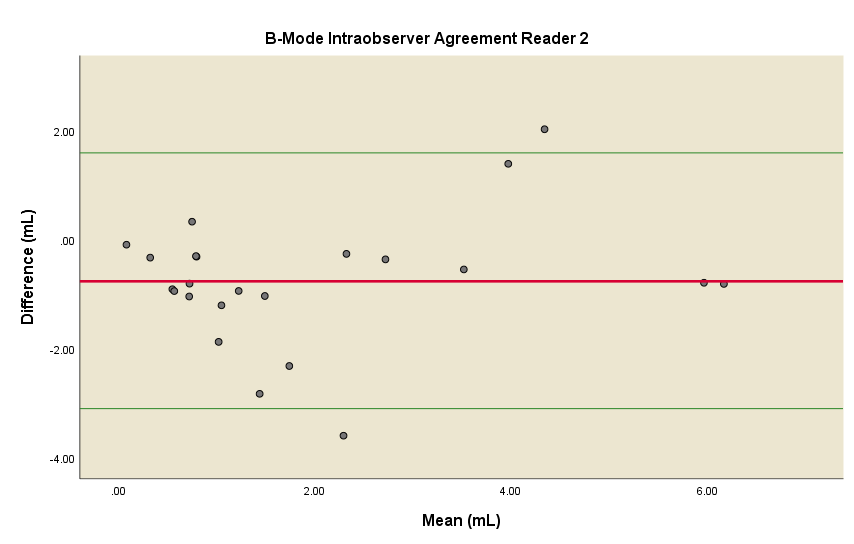


**Supplementary Figure 4.** Reader 2 B-mode.
