# Supplementary figures and images for: Reproducibility of Ablated Volume Measurement Is Higher with Contrast-Enhanced Ultrasound than with B-Mode Ultrasound after Benign Thyroid Nodule Radiofrequency Ablation—A Preliminary Study
Source: J Clin Med. 2020 May 16;9(5):1504. doi: 10.3390/jcm9051504 (PMC7291258; doi:10.3390/jcm9051504)

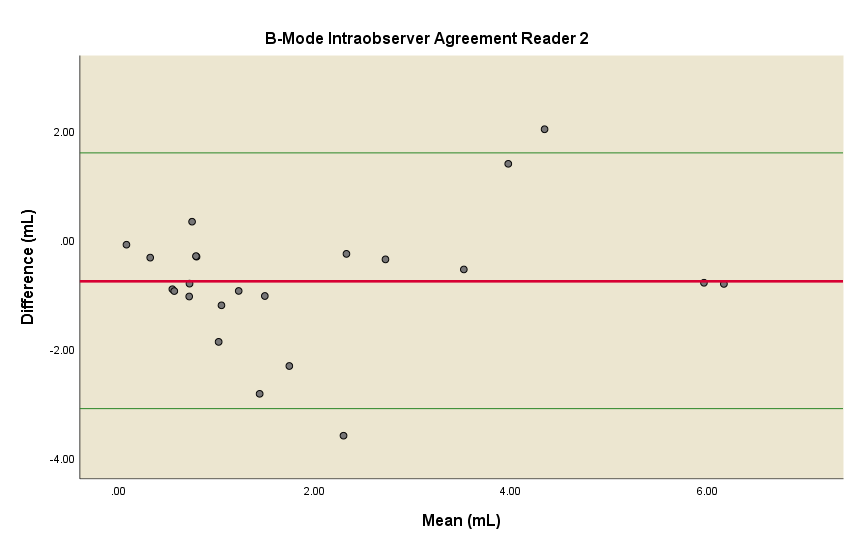

Supplement: Supplementary file 1 [file jcm-09-01504-s001.zip › Supplementary Plots/Supplement_4.tif]

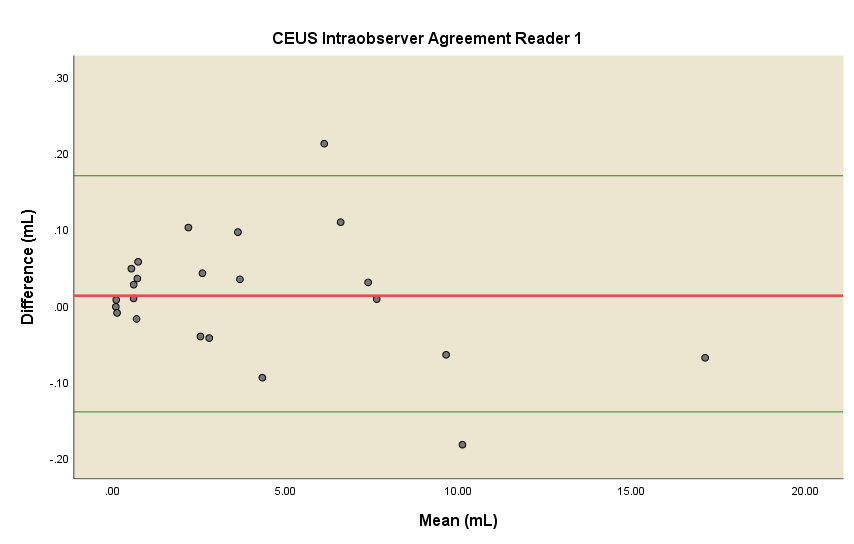

Supplement: Supplementary file 1 [file jcm-09-01504-s001.zip › Supplementary Plots/Supplement_1.tif]

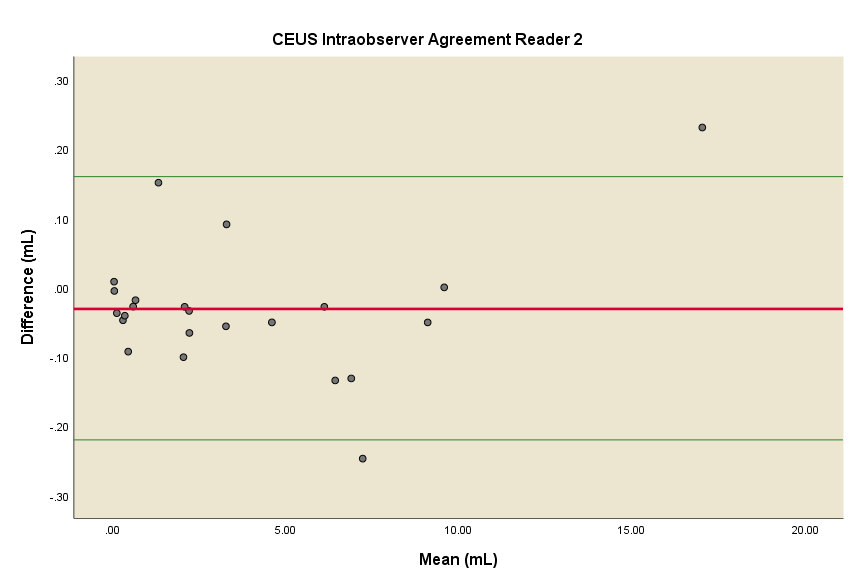

Supplement: Supplementary file 1 [file jcm-09-01504-s001.zip › Supplementary Plots/Supplement_3.tif]

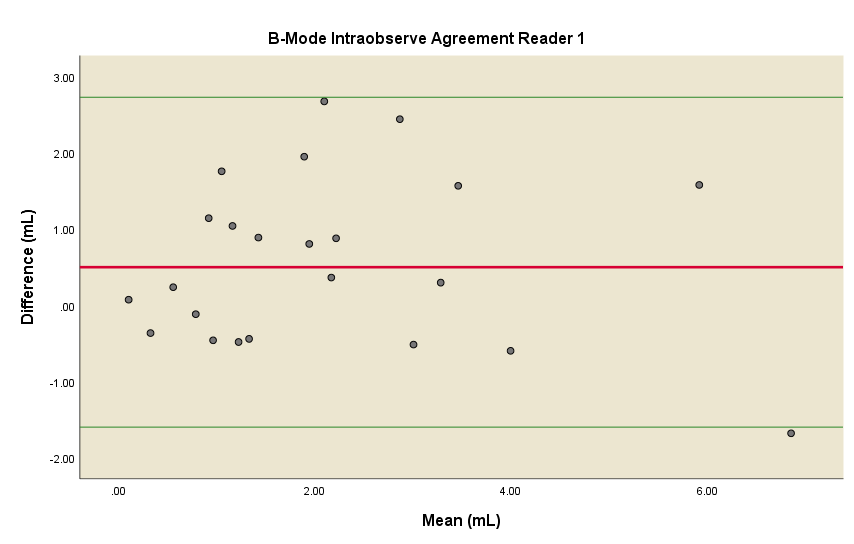

Supplement: Supplementary file 1 [file jcm-09-01504-s001.zip › Supplementary Plots/Supplement_2.tif]
